# Editorial: Benefits and risks of agonist triggering strategies

**DOI:** 10.3389/fendo.2024.1538412

**Published:** 2024-12-23

**Authors:** Jan Tesarik

**Affiliations:** MARGen Clinic, Granada, Spain

**Keywords:** GnRH agonist, GnRH antagonist, controlled ovarian stimulation, embryo viability, uterine receptivity, early pregnancy

## 1 Introduction

Since many years, the GnRH analogues GnRHa and GnRHant are used alternatively for preventing premature LH surge and ovulation in controlled ovarian stimulation protocols (1). Recently, GnRHa is also used in GnRHant-controlled cycles as an alternative to human chorionic gonadotropin (hCG) to trigger final oocyte maturation and ovulation (2). The use of these GnRH analogues simplifies the ovarian stimulation protocol and reduces the risk of ovarian hyperstimulation syndrome (1, 2). After initial warning voices, based on animal experiments and suggesting that GnRH and its analogues may interfere with the early pregnancy through their action on the corpus luteum and the uterus (3), these fears were not substantiated in clinical practice (1, 2). However, some doubts may still persist. This Research Topic addresses this question, in addition to bringing together other new data relative to the efficacy and safety of controlled ovarian stimulation protocols.

## 2 The main points of individual contributions

This series includes 9 original research articles, focusing on GnRHa effects on embryo viability, uterine receptivity and early pregnancy, as well as some other new aspects of controlled ovarian stimulation in general. In this section, they are presented in a chronological order of publication in the Journal. Pang et al. investigated into the relationship between serum luteinizing hormone (LH) concentration on the day of the beginning of GnRHant administration during ovarian stimulation for conventional *in-vitro* fertilization (IVF) or intracytoplasmic sperm injection (ICSI), on the one hand, and laboratory indicators and clinical outcomes on the other hand. They report a significant positive correlation between LH concentration on the antagonist administration day and the numbers of oocytes retrieved, of two-pronucleated embryos and of blastocysts. In a propensity score-matched study, Zhang et al. explored the cycle characteristics and pregnancy outcomes of progestin-primed ovarian stimulation using fixed versus degressive doses of medroxyprogesterone acetate (MPA) in conjunction with letrozole (LE) in infertile women. They did not find any significant differences in the incidence of premature LH surge, the number of oocytes retrieved, the number of top-quality embryos, clinical pregnancy rate, cumulative live birth rate or fetal malformation rate between the two groups, while the combination of a degressive MPA dose with LE proved effective in reducing total MPA dose. A parallel, open-label randomized trial by Li et al., including 245 women, examined the usefulness of intramuscular injection of human chorionic gonadotropin (hCG) after embryo transfer as luteal phase support in artificial cycle frozen-thawed embryo transfer attempts failed to find any improvement of clinical outcomes in the hCG group. A retrospective cohort study by Cao et al. compared pregnancy outcomes in fresh IVF/ICSI cycles in 294 women who had recovered from COVID-19 infection with those of 631 women who had not been infected. No substantial evidence was found between the two groups. Li et al. used transcriptome profiling to analyze the impact of using GnRHa as ovulation trigger on embryo implantation and early pregnancy in superovulated mice. Their findings suggest that a combination of ovarian stimulation and GnRHa trigger impair embryo implantation in mice, presumably due to changes in endometrial gene expression, namely concerning the genes responsible for endometrial remodeling, ion transport, and immune response. A retrospective cohort study conducted by Cao et al. compared live birth rates after IVF/ICSI in 924 treatment cycles using GnRHant original reference product Cetrotide with those in 1984 cycles using a generic GnRHant (Ferpront). No differences between the attempts using either of the two preparations were detected. Hao et al. compared retrospectively clinical outcomes of frowen-thawed embryo transfer in patients prepared with the combined use of hormone replacement therapy (HRT) and GnRHa (leuprorelin) with those achieved with HRT (estradiol valerate) alone. Clinical pregnancy and implantation rates achieved with the combined (HRT + GnRHa) protocol were higher as compared with HRT alone. Luo et al. used logistic regression analysis to identify the risk factors for empty follicle syndrome (EFS). They further analyzed IVF cycles of patients with EFS and performed long-term follow-up of those who had got pregnant until live birth was achieved. They identified polycystic ovary syndrome as an independent risk factor for EFS and showed that repeated instances of EFS are associated with poor reproductive prognosis. Finally, Hsu et al. investigated the correlation between the ovarian sensitivity index (OSI) and clinical parameters in GnRHa and GnRHant cycles. Serum anti-Mullerian hormone, cycle 2 follicle stimulating hormone (FSH), LH and estradiol concentrations, numbers of large follicles, fertilization rate, and the incidence of premature LH surge were positively correlated with the OSI. The GnRHa and GnRHant protocols did not differ as to the incidence of premature LH surge and ovulation, but higher numbers of mature oocytes and good-morphology embryos were obtained in the GnRHa cycles.

## 3 Synthetic view and conclusions

Taken together, the data presented in this Research Topic touch various aspects of GnRHa and GnRHant effects on assisted reproduction outcomes. In addition, data unrelated to these two substances but important for improving controlled ovarian stimulation protocols are also included. Most of data presented support the inclusion of GnRHa and GnRHant in these protocols.
